# Factors affecting access to and utilisation of intravenous iron to treat anaemia in pregnancy in Zomba, Malawi: a qualitative study

**DOI:** 10.1136/bmjgh-2025-019886

**Published:** 2026-05-07

**Authors:** Elisabeth Mamani-Mategula, Hana Sabanovic, Naomi Von Dinklage, Ebony Verbunt, Khic-Houy Prang, Effie Chipeta, Lucinda Manda-Taylor

**Affiliations:** 1 Department of Health Systems and Policy, Kamuzu University of Health Sciences, Blantyre, Malawi; 2 School of Population and Global Health, Centre for Health Policy, The University of Melbourne, Melbourne, Victoria, Australia; 3 Population Health and Immunity Division, Walter and Eliza Hall Institute of Medical Research, Melbourne, Victoria, Australia

**Keywords:** Maternal health, Qualitative study, Health Services Accessibility

## Abstract

**Background:**

Anaemia in pregnancy significantly increases the risk of maternal and neonatal complications. Oral iron supplementation, the standard treatment, is often poorly tolerated and associated with low adherence. Intravenous iron presents a rapid alternative, and we examined the factors influencing access to and utilisation of intravenous iron in treating anaemia in pregnancy within Malawi’s primary healthcare system.

**Methods:**

We conducted a qualitative study embedded with a clinical trial. We collected data through in-depth interviews (n=16) and focus group discussions (n=3) with pregnant women enrolled in the trial, those who withdrew and their husbands and caregivers. Interviews were audio-recorded, transcribed and analysed using a reflexive thematic approach in NVivo 12. Themes were mapped onto the supply and demand domains of the Patient-Centred Access to Healthcare framework.

**Results:**

Barriers to access and utilisation operated across both supply-side and demand-side domains. On the supply side, limited service awareness, logistical constraints and unclear provider communication reduced engagement with intravenous iron. On the demand side, pregnant women’s knowledge about anaemia, cultural norms, personal beliefs and social support shaped decision-making and ability to access care. Facilitators across both domains included patient-centred service delivery, clear and responsive provider communication and family or peer involvement in decision-making, which enhanced trust, understanding and uptake.

**Conclusion:**

In conclusion, our study demonstrates that patient-centred approaches are critical for improving health literacy, and strong health systems are essential for the effective delivery of intravenous iron intervention. Addressing supply and demand barriers is vital for optimising uptake of intravenous iron interventions, improving health system performance and ensuring equitable access to antenatal care interventions, ultimately improving the health outcomes for mother and child.

**WHAT IS ALREADY KNOWN ON THIS TOPIC**

- Intravenous iron has been established as an effective intervention for managing anaemia in pregnancy, with successful implementation widely documented in high-income countries.
- Assessing its implementation in low- and middle-income countries is essential to understand its accessibility, utilisation and overall impact on maternal health.

**WHAT THIS STUDY ADDS**

- This study examines intravenous iron implementation from both supply-side and demand-side perspectives, capturing barriers and facilitators in Malawi’s primary healthcare system.
- Insights were gathered from pregnant women and their husbands and caregivers to identify real-world challenges on how cultural norms and beliefs shape health-seeking behaviour and practices, contributing to our understanding of how to tailor health interventions to cultural contexts. Additionally, it highlights facilitators for health system improvements to align with patient-centred care needs.

**HOW THIS STUDY MIGHT AFFECT RESEARCH, PRACTICE OR POLICY**

- The Malawian health system currently lacks clear evidence to guide intravenous iron adoption, emphasising the need for research to inform national policies and clinical guidelines for anaemia management.
- Findings from this study will help policymakers and healthcare providers develop more effective maternal health strategies, bridge knowledge gaps in LMICs and improve service delivery.

## Introduction

 Anaemia in pregnancy is a major global health concern, affecting approximately 40% of pregnant women worldwide.^1^,^3^ The burden is highest in Sub-Saharan Africa, where prevalence exceeds 45%, and remains substantial in Malawi, with nearly 40% of pregnant women affected by anaemia.^4 5^ Anaemia in low- and middle-income countries (LMICs) arises largely from iron deficiency, infections such as malaria and HIV, inadequate dietary intake and limited access to timely antenatal care.^1 6^

Symptoms commonly include fatigue, dizziness, headache and shortness of breath.^1 7 8^ Untreated anaemia increases the risk of maternal mortality, preterm birth, low birth weight and long-term developmental deficits in children.^6 9^ Addressing anaemia burden in pregnancy is central to the WHO Global Nutrition Targets for 2030, which aim to halve anaemia among women of reproductive age and align with Sustainable Development Goals 2 and 3.^7 8^ Although oral iron supplementation of 120 mg daily intake is the standard treatment,^10^ its impact is constrained by poor adherence, gastrointestinal side effects, inconsistent supply and slow replenishment of iron stores.^11^,^13^ These limitations highlight the need for more effective alternatives.

Intravenous iron, especially modern formulations like ferric carboxymaltose (FCM), enables rapid iron repletion with better tolerability.^14^ Intravenous iron use has expanded rapidly in high-income countries^15^,^20^ due to its availability, strong clinical guidelines, well-resourced health systems capable of delivering infusions safely and a growing preference for faster, more convenient treatment options.^15 20 21^ However, utilisation remains limited in low-resource settings like Malawi, highlighting the need to understand contextual barriers and opportunities for implementing intravenous iron therapy. Since effective utilisation depends not only on individuals’ willingness to seek care but also on their ability to reach and engage with care, understanding access is critical in determining uptake. To address this gap, we examined supply and demand factors affecting access to and utilisation of intravenous iron for the treatment of anaemia in pregnancy within Malawi’s primary healthcare setting.

## Methodology

### Overall study setting

This qualitative study was conducted within the broader Randomised Controlled Trial of the Effect of intraVenous iron on Anaemia in Malawian Pregnant women in the Third Trimester (REVAMP-TT) and its nested Implementation Science programme (REVAMP-IS). REVAMP-IS began earlier than the trial, in February 2021, because its other purpose was to conduct the formative research and community engagement activities that informed the design of the REVAMP-TT trial. REVAMP-IS ran from February 2021 to December 2023. In contrast, the REVAMP-TT trial itself was implemented from September 2021 to December 2023. Embedding REVAMP-IS within REVAMP-TT ensured integrated evidence on both effectiveness and implementation, providing critical insights for the potential national-scale-up of intravenous iron therapy in Malawi. This hybrid design helped identify real-world implementation challenges early, enhancing the intervention’s relevance and readiness for future adoption.

### REVAMP-TT trial (effectiveness component)

The trial evaluated whether a single intravenous iron infusion of FCM was more effective than oral iron in treating moderate or severe anaemia in the third trimester and improving maternal and child health outcomes.^22^ The trial was conducted across eight public primary healthcare facilities, four urban (City Clinic, Sadzi, Matawale, Naisi) and four rural (Domasi Rural Hospital, Bimbi, Likangala, Lambulira). The trial was coordinated through the Training and Research Unit of Excellence and delivered in collaboration with government healthcare providers, reflecting real-world conditions. The REVAMP-TT trial findings have been reported elsewhere.^23^

### REVAMP IS

REVAMP-IS examined how intravenous iron therapy could be designed, delivered, accepted and scaled in Malawi’s primary healthcare system. A protocol paper detailing this research programme was already published.^24^ The programme had three phases: phases 1 and 2 involved identifying potential barriers and facilitators through the key informants and using an experience-based codesign approach to develop implementation strategies for the trial^25^ and phase 3 is the current study.

### Study approach

Through the end user’s lens, we evaluated the demand-side and supply-side factors affecting access to and utilisation of intravenous iron therapy, guided by the Patient-Centred Access to Healthcare (PCAH) framework.^26^ In this context, access refers to the ability to reach and receive healthcare promptly to achieve the best possible health outcome while utilisation reflects whether individuals recognise the need for care, seek it and are able to obtain.^27 28^

The PCAH framework guided the development of data collection tools and analysis, allowing us to map barriers and facilitators from a patient-centred perspective, considering the interaction between supply and demand. We conducted in-depth interviews (IDIs) with pregnant women who received intravenous iron and focus group discussions (FGDs) with the pregnant women and their caregivers (husbands, mothers and mothers-in-law). Using a single interview topic guide, from the demand side, we explored participants’ understanding of anaemia and intravenous iron (ability to perceive), willingness to seek care and social or cultural influences (ability to seek), travel and access challenges (ability to reach), and experiences with intravenous iron, including discomfort and satisfaction (ability to engage). From the supply side, we explored health system processes (approachability), service delivery, communication, staff interactions (acceptability), resource availability (availability and accommodation) and the perceived effectiveness and quality of intravenous iron therapy. Table 1 outlines the PCAH framework dimensions as described by Levesque *et al*.^26^

**Table 1:** Definitions of the supply and demand domains of the PCAH framework

	Domain	Definition
Supply side	Approachability	Refers to how easily a health system or service can be accessed by individuals, including factors like geographic location and the presence of barriers to entry.
	Acceptability	The extent to which health services meet the expectations, cultural beliefs and preferences of individuals or populations, influencing their willingness to use the services.
	Availability and accommodation	The availability of healthcare services and resources, and their ability to accommodate the needs of individuals in terms of timing, location and service offerings.
	Appropriateness	The relevance and quality of the services provided in meeting the health needs of individuals or populations.
Demand side	Ability to perceive	The ability of individuals to recognise and understand their health needs and the potential for care.
	Ability to seek	The ability of individuals to actively look for and initiate contact with the healthcare system when a health need arises.
	Ability to reach	The ability of individuals to physically access healthcare services, considering barriers such as transportation, cost and physical proximity.
	Ability to engage	The ability of individuals to actively participate in the healthcare process, including communication with providers and adherence to treatment plans.

PCAH, Patient-Centred Access to Healthcare.

### Participant sampling

#### In-depth interviews

For the IDIs, we included pregnant women (n=16) who were randomly selected from a total of 420 participants listed in the REVAMP-TT trial register, ensuring two participants from each of the eight study sites. The trial coordinator provided their identification numbers and contact information, and we invited them by phone and home visit. We included pregnant women who had received intravenous iron treatment and were over 18 years old. To allow for possible decline, 19 women were selected, 16 agreed to participate, while 3 declined because they had withdrawn from the trial.

#### Focus group discussions

After completing the IDIs, we conducted three FGDs to triangulate our data collection methods and capture broader perspectives. For the FGDs, we used a mixed sampling approach combining random selection with purposive selection. This approach enabled us to include key decision-makers who influence pregnant women’s health-seeking behaviour. The FGDs fostered a collaborative dialogue, providing a deeper understanding of the social and cultural factors influencing intravenous iron use and ensuring a wide range of perspectives for more comprehensive feedback. This approach enhanced the validity of our findings and helped minimise research biases. In total, we invited 35 participants: 4 declined and 31 agreed to participate. The FGDs were organised as follows:

- **First FGD (n=10):** pregnant women randomly selected from the REVAMP-TT trial register who were still enrolled and actively participating in the trial.
- **Second FGD (n=9):** consisted of pregnant women who were still participating in the trial, randomly selected (n=3), and women who had withdrawn from the trial, purposively selected (n=3). The women who had withdrawn were asked to bring their husbands (n=3).
- **Third FGD** (**n=12):** pregnant women who were still enrolled in the trial (n=6), together with their husbands (n=3) and other caregivers (n=3).

### Data collection

Having received training in qualitative research approaches and data collection, we (EM-M, GK, WS) conducted in-depth interviews with pregnant women at the health facilities where they received intravenous iron. We followed a structured interview guide. The IDIs lasted approximately 45 min to 1 hour. After conducting the IDIs, we held the FGDs. Two facilitators led the FGDs: one (EM-M) facilitated the discussion, while the other (GK) took notes and recorded. Each FGD lasted between 1 hour and 30 min and 2 hours. We invited the participants to a nearby health facility. We used a designated private room at the health facility for the interviews to ensure a calm, private environment in which they could express their views. An interview guide with questions and prompts was used to balance flexibility in the conversation with adherence to the structured data-collection framework. As preferred by the participants, all FGDs and IDIs were conducted in Chichewa, the local language. All interviews were recorded on a digital recorder, and we made field notes throughout the data collection process, which were used for debriefing sessions. We recruited participants for the study who took part in the IDIs and FGDs from 12 September 2022 to 24 February 2023 and collected data within the same period. The trial team had designated nurses and coordinators to implement the intervention, while the implementation science team only supported community engagement activities and informed the trial’s design. Therefore, interviewers and focus group facilitators were not involved in trial implementation and did not influence participant responses. All participants received US$10 in compensation, following the National Health Sciences Research Committee in Malawi’s recommendations for human subjects participating in research.^29^

### Data analysis

Two research assistants (WS, GK) transcribed all the interviews verbatim into English, and EM-M crosschecked the transcripts for data curation and to verify their accuracy against the audio recordings. During data analysis, we employed reflexive thematic analysis, a flexible approach that iteratively identifies patterns of meaning to enable in-depth engagement across the dataset.^30^ First, we (EM-M, HS) familiarised ourselves independently with the dataset by reading the transcripts multiple times to gain an in-depth understanding of the content. During this process, we inductively generated initial codes and potential themes. These preliminary codes informed the development of an initial codebook, which included descriptions and definitions for each code and theme. We then refined the initial codebook through iterative discussions to reach consensus on the final version. Using this final codebook, EM-M coded all the transcripts to identify detailed codes and generate new codes iteratively until no additional themes or insights emerged from the data. We used NVivo 12 to organise, manage and facilitate the comparison and synthesis of the data, ensuring a systematic and comprehensive analysis. We then reviewed, refined and clustered the emerging themes and deductively mapped them to the PCAH framework. Final themes were interpreted in relation to the study objectives and outcomes with representative quotes from the participants. The combined inductive and deductive approach used ensured the analysis was data-driven and grounded in a structured framework.

### Researcher’s positionality and reflexivity

This paper reflects the researcher’s background in health research and social sciences, with extensive experience implementing health interventions in LMICs, particularly Malawi. The study adopts a community-driven, participatory approach grounded in advocacy, social justice and equity. This positionality shaped the study design, prioritising methods that amplify the voices of pregnant women and their families as key decision-makers. Reflexivity was crucial to ensuring the findings remained participant-centred and minimised bias. The study highlights the socio-cultural influences on healthcare access and emphasises context-specific solutions to overcome barriers. Findings were framed to promote equitable health practices and inform evidence-based recommendations for improving intravenous iron utilisation.

### Transparency and credibility

The first author, EM-M, piloted the interview guide with two pregnant women from the REVAMP-TT trial who were not part of the study. Feedback led to refinements in the tool, developed with two experienced research assistants (GK, WS). Changes included rewording difficult questions and adding probes for deeper insights. Daily debriefs over 6 months allowed the team to review and adjust questions, ensuring comprehensive data collection and improving study credibility through data triangulation from the FGDs and IDIs.

## Results

We conducted 16 IDIs with 2 participants per site and 3 FGDs with 31 participants across the eight sites. Table 2 summarises the total number of participants who consented and were interviewed for IDIs and FGDs.

**Table 2:** Summary of the participants recruited

Participant group		Number of participants			
		IDIs	FGD1	FGD2	FGD3
1	Pregnant women who received intravenous iron	16	10	3	6
2	Pregnant women who received intravenous iron but withdrew from REVAMP-TT trial			3	
3	Husbands to pregnant women who withdrew from REVAMP-TT trial			3	
4	Husbands to pregnant women who received intravenous iron				3
5	Mothers and mothers-in-law of the pregnant women who received intravenous iron				3
Total number of participants		16	10	9	12

FGD, focus group discussion; IDIs, in-depth interviews; REVAMP-TT, Randomised Controlled Trial of the Effect of intraVenous iron on Anaemia in Malawian Pregnant women in the Third Trimester.

Table 3 presents the demographic characteristics of participants, including pregnant women, their husbands, and mothers or mothers-in-law who attended the IDIs and the FGDs. In our study, we examined both demand-side and supply-side factors influencing access to and utilisation of intravenous iron for treating anaemia in pregnancy within Malawi’s primary healthcare setting. Our findings, detailed in table 4, are mapped to the PCAH framework.

**Table 3:** Participants demographic characteristics

Variables	IDI	FGD		
	Pregnant women	Pregnant women	Husbands	Mothers and mothers-in-law
Age				
18–29	8	15	1	–
30–39	5	7	4	–
40–49	3		1	
50–59	–	–	–	3
Marital status				
Married	11	21	6	2
Unmarried	5	1	–	–
Divorced/widowed	–	–	–	1
Education				
Illiterate	1	–	–	2
Primary	8	17	2	1
Secondary	7	5	4	–
Tertiary	–	–	–	–
Occupation				
Employed	–	–	1	–
Unemployed	8	3	–	–
Business	5	8	2	–
Farmer	3	11	3	3
Health facility				
Lambulira	2	2	1	
Matawale	2	2		1
Naisi	2	2	1	
Sadzi	2	3		1
City Clinic	2	3		1
Domasi	2	3	1	
Likangala	2	4	2	
Bimbi	2	3	1	
Religion				
Christianity	11	13	2	1
Islam	5	9	4	2

FGD, focus group discussion; IDI, in-depth interview.

**Table 4:** A summary of the findings highlighting the supply and demand barriers and facilitators

Category	Barriers	Facilitators
Supply side	Approachability Lack of transparency specific to REVAMP-TT trial processes and procedures that discouraged the uptake of intravenous iron Limited awareness of intravenous iron in the community	Approachability Availability of clear information about anaemia and antenatal care services
		Acceptability Pregnant women were not pressured to receive intravenous iron
	Availability and accommodation Distance as a barrier to accessing antenatal care services where intravenous is offered Long waiting times for procedures and intravenous iron administration	Availability and accommodation Flexible and accessible antenatal care services for intravenous iron administration
	Appropriateness Pregnant women’s non-compliance with health facility appointments	Appropriateness Perceived effectiveness and benefits of intravenous iron Healthcare providers’ interpersonal skills and quality of care
Demand side	Ability to perceive Health beliefs, myths and misconceptions about intravenous iron	Ability to perceive Health literacy Awareness of signs, symptoms and consequences of anaemia
	Ability to seek The impact of cultural norms and beliefs such as concealing pregnancy Social values and sense of autonomy influencing decision-making Limited peer support	Ability to seek Peer support facilitated informed healthcare decision-making
	Ability to reach Mobility concerns Lack of social and financial support from husbands	Ability to reach Available social and financial support from family or husband (transportation, food provision)
	Ability to engage Physical discomfort during intravenous iron administration	Ability to engage Caregiver support (emotional and practical support from husband or mother/mother-in-law)

REVAMP-TT, Randomised Controlled Trial of the Effect of intraVenous iron on Anaemia in Malawian Pregnant women in the Third Trimester.

### Supply-side barriers and facilitators to intravenous iron utilisation

#### Supply-side barriers

We identified five key themes across three domains of supply-side barriers that could hinder access to and utilisation of intravenous iron.

##### Approachability: limited awareness of intravenous iron in the community

Many pregnant women reported first learning about intravenous iron at the health facility, making it hard to accept. They suggested using radio, posters, market days and community campaigns to raise awareness and encourage the use of intravenous iron.

Of course, you said that you have already done community awareness. But I will advise that you should continually go to the communities and engage with the local leaders to spread the information about the IV iron intervention because women are still getting pregnant day by day, and they may not be aware of the IV iron intervention; you can also use radio programs as they do on Mzati radio. (FGD2, Husband#3)

##### Availability and accommodation: distance as a barrier to accessing antenatal care services where intravenous is offered

Although intravenous iron was available at the health facility, many pregnant women and caregivers delayed or avoided seeking antenatal care services due to the long distances, often requiring up to 2 hours on foot. This led to missed opportunities to receive intravenous iron at an earlier stage.

I had just moved to my mother-in-law’s place so that I could be close to the health facility. When I came for my first visit to register, they found I had anaemia and needed an intravenous iron infusion. I had not been attending antenatal care before because my husband and I live very far away, near Mozambique. (IDI, Pregnant woman#12)

##### Availability and accommodation: long waiting times for procedures and intravenous iron administration

A significant challenge identified by many pregnant women was the prolonged waiting times at the health facilities, which hindered their timely access to intravenous iron. Although routine antenatal procedures, registration, vital checks and anaemia screening were similar for all women, those receiving intravenous iron experienced additional delays specific to the intervention. Unlike women receiving oral iron, who were able to leave immediately after receiving their tablets, intravenous iron recipients were required to remain for infusion and post-infusion monitoring, extending their visit by about an hour. As a result, total time spent at the facility could reach 6 hours, creating a substantial burden. Participants described this prolonged stay as discouraging and a barrier to timely intravenous iron administration.

The waiting time was indeed long. I arrived at 8 o’clock and left around 2 o’clock. On top of that, I was starving because I hadn’t eaten that morning. The process was long; before the treatment, the nurses gave information about the IV iron intervention and allowed us to ask questions. (IDI, Pregnant woman#3)

##### Appropriateness: pregnant women’s non-compliance with appointments

Many pregnant women reported non-compliance with antenatal care appointments as a barrier to timely access to intravenous iron. Some pregnant women were requested to return home to seek their husband’s permission before receiving intravenous iron and were given a follow-up date to return. However, many did not return for intravenous iron, resulting in delays or missed opportunities.

I honestly did not consult my husband because I was looking out for myself. I just agreed to receive the IV iron treatment. But some of my friends were hesitant and wanted to consult first because of the misconceptions they were hearing, and they never returned. (IDI, Pregnant woman#4)

Additionally, delayed first visits and the perceived burden of frequent appointments, combined with competing household responsibilities and childcare, led some women to miss or postpone scheduled visits, which in turn delayed or prevented timely intravenous iron therapy.

Sometimes you may think your pregnancy is still settling, so you decide to wait a bit. Meanwhile, time is passing. At six months, you start feeling tired and thinking you should rest and stay home, even as you have to take care of your other children. That’s how we miss it [the screening and treatment for anaemia]. (IDI, Pregnant woman#14)

### Supply-side facilitators

We identified five themes across the four supply-side domains of the PCAH framework that facilitate the use of intravenous iron for the treatment of anaemia in pregnancy.

#### Approachability: availability of clear information about anaemia and antenatal care services

Many pregnant women reported that healthcare providers at the facilities provided clear information and explanations about intravenous iron treatment. In addition, educational materials on anaemia in pregnancy, created under REVAMP-IS and available at the facility, significantly improved their understanding of anaemia and supported informed decision-making about intravenous iron therapy. Pregnant women indicated that these materials simplified their understanding of anaemia-related challenges during pregnancy and encouraged them to receive intravenous iron.

Whenever I attend an antenatal care visit, the nurse provides all the necessary information and explains what we should expect. She educates us about the dangers of anaemia, how it can affect unborn babies, and the importance of treating it. (IDI, Pregnant woman#12)

In addition, many pregnant women and their caregivers stressed that providing intravenous iron therapy as part of antenatal care services at health facilities strengthened their trust in healthcare services. Offering intravenous iron alongside existing antenatal care services reinforced their confidence in the overall quality and reliability of the healthcare system.

We trust the services that are offered by the nurses we already know at the health facility; they are the ones who help us here in the communities, and there is no way researchers would come directly to us without passing through them. (FGD2, Mother-in-law#4)

#### Acceptability: pregnant women were not pressured to receive intravenous iron

Pregnant women consistently reported that they felt no pressure or coercion when deciding whether to receive intravenous iron. Instead, they described a process in which health workers provided clear explanations, sought informed consent and engaged with them respectfully, taking into account their background, literacy level and emotional needs. This supportive, non-judgmental communication enhanced women’s comfort and increased their willingness to receive intravenous iron.

It was easy for me to accept because I was briefed, and they [nurses] gave me a form with all the information about the trial. It explained some of the treatment’s side effects, such as feeling weak. So, the nurse assured me that if I ever experience such, I should not be worried and assured that they would look after me if anything goes wrong. (IDI, Pregnant woman#4)

#### Availability and accommodation: flexible and accessible antenatal care for intravenous iron administration

Pregnant women appreciated the accessibility of trial facilities during antenatal care visits and valued the opportunity to discuss follow-up care with healthcare providers. Flexible scheduling allowed women to plan visits in consultation with their husbands, reducing stress and ensuring family involvement. This approach helped to address concerns and misconceptions about intravenous iron administration, making the treatment experience more reassuring and manageable.

When I found out about my condition, I was filled with worry because I had heard a lot of bad stories regarding this [intravenous iron] treatment. I hear that the health facility takes a lot of blood and sells it. So I went home and told my husband. At that time, I had swollen feet. So, my husband was like, “You have swollen feet, and you look pale because you have low blood levels”, so he escorted me the next day to the health facility where I received the [intravenous iron] treatment. Then I noticed that everything that people were talking about, I never experienced. (FGD1, Pregnant woman#4)

#### Appropriateness: perceived effectiveness and benefits of intravenous iron

Pregnant women receiving intravenous iron reported significant health improvements, including reduced anaemia symptoms such as headache, dizziness and fatigue within days. They appreciated the single-dose treatment, which avoids daily oral supplements and eliminates gastrointestinal side effects, making it a more comfortable option for managing anaemia in pregnancy.

Yes, I approve of the IV iron intervention. After receiving the IV iron treatment, I noticed a significant change: unlike the [oral iron] tablet one, I felt nauseous and dizzy and saw no change. In addition, IV iron is taken once, which I like, unlike the iron tablet, which you are supposed to take daily. (IDI, Pregnant woman#1)

#### Appropriateness: healthcare providers’ interpersonal qualities and skills

Many pregnant women reported that the intravenous iron intervention was appropriately delivered. They appreciated the dedication of the skilled healthcare providers who provided the service. They expressed no concerns about the treatment they received or about being treated by male or female healthcare providers. Pregnant women emphasised that the intervention was well suited for their needs, contributing to improvements in their health and overall well-being.

There was nothing scary, as people used to say. The nurses were very friendly and allowed me to ask any questions. They did not even force me to participate. It was my own choice after learning of my condition and the possible IV iron treatment. (IDI, Pregnant woman#8)

### Demand-side barriers and facilitators to intravenous iron utilisation

#### Demand-side barriers

Within the PCAH framework’s four domains, we identified four key demand-side barriers to the use of intravenous iron for managing anaemia in pregnancy.

##### Ability to perceive: myths and misconceptions about intravenous iron

Participants reported that widespread myths and misconceptions significantly shaped how pregnant women perceived intravenous iron therapy. Misinformation circulated both within antenatal care groups and in the broader community, leading some women to distrust medical advice. These misconceptions included fears that intravenous iron could cause congenital abnormalities, malnutrition or miscarriage or that blood taken during treatment would be sold. As a result, some pregnant women rejected intravenous iron treatment or opted for traditional remedies such as *chidede* (hibiscus) to manage anaemia. These inaccurate beliefs limited women’s ability to recognise the benefits and safety of intravenous iron therapy correctly.

Ok, what happens is that when we come to attend the antenatal care, some women in the group start spreading rumours that the IV iron is suspicious, we will give birth to abnormal babies, and we don’t know where they take the blood to, so we should not agree to receive it when they diagnose us with anaemia. As a result, people start agreeing to their idea, and others leave. (FGD3, Pregnant woman#6)

##### *A*bility to seek: the impact of cultural norms and beliefs

Participants also highlighted how cultural beliefs and social norms influenced pregnant women’s willingness and timing in seeking antenatal care and intravenous iron treatment. Many women concealed their pregnancies and delayed accessing antenatal care services to avoid becoming the subject of community gossip or attracting perceived ill will from others. This reluctance to present early at health facilities resulted in late diagnosis of anaemia and reduced opportunities for timely intravenous iron intervention. These cultural pressures, therefore, constrained women’s ability to actively seek and initiate appropriate care.

We wait until we can visibly confirm the pregnancy. Telling everyone that you’re pregnant not only makes you the talk of the community but also exposes you to others who may wish you bad luck. (FGD2, Mother Participant#7,)

##### Ability to reach: mobility concerns and lack of social and financial support from husbands

Many pregnant women reported that the physical strain of late pregnancy, coupled with long distances to the health facility, made accessing care challenging. As their pregnancies progressed, walking became difficult, and some chose to delay or forgo visits. In the trial, where intravenous iron was administered in the third trimester, this mobility issue further hindered the timely access to intravenous iron.

Walking long distances to the health facility is exhausting, especially when you are heavily pregnant. It’s even harder when your husband isn’t available, you almost wish he could experience the challenges we face so he could understand what we go through. (FGD1, Pregnant woman#7)

Many pregnant women reported that financial constraints, particularly the lack of support from their husbands, significantly hindered their access to antenatal care and timely intravenous iron treatment. They noted that they often could not afford transport to the health facility for antenatal care appointments, relying on motorbikes or ‘kabaza’, the primary means of transport in rural Malawi. They said that when faced with the choice between attending appointments and buying food for their families, many opted to skip the appointment, resulting in missed opportunities for anaemia screening and intravenous iron administration.

I separated from my husband, and there was no one to support me. Even if I tried to reason with him that this child belongs to all of us, he has never spent a single penny on this pregnancy. So, because I struggle to make ends meet, I feel the money I find from casual jobs should cater for my food, instead of antenatal care visits. (IDI, Pregnant woman#14)

Furthermore, pregnant women reported that the lack of support from their husbands, either due to work commitments or financial constraints, delayed their healthcare decisions to receive intravenous iron and further postponed receiving necessary treatment.

Unfortunately, my husband was not available when I attended my antenatal care visit and was diagnosed with anaemia. I had to decline the [intravenous iron] infusion at that moment because I wanted to discuss it with him first. The child is our responsibility, and if I were to lose the pregnancy due to the medication from the health facility, my husband would hold me solely accountable. (IDI, Pregnant woman#6)

##### Ability to engage: physical discomfort during intravenous iron administration

Pregnant women also reported experiencing physical discomfort due to the intravenous iron administration, such as pain during cannulation, headaches lasting several days and feelings of hunger due to the lengthy procedures, all affecting their physical comfort.

I did not like having the cannula on my arm; it was painful and uncomfortable. The waiting time was also long, and I felt hungry because I had not eaten anything since morning. (IDI, Pregnant woman#8)

### Demand-side facilitators

We also identified five key themes from the four demand-side domains of the PCAH framework that facilitate the use of intravenous iron to treat anaemia in pregnancy.

#### Ability to perceive: increased health literacy

Pregnant women described how improved understanding of anaemia and its causes, consequences and available treatments enhanced their ability to perceive their health needs accurately. Health literacy enabled them to recognise the seriousness of anaemia and the potential benefits of intravenous iron, helping them distinguish reliable medical information from community myths or traditional and religious healers. This improved knowledge strengthened their confidence in biomedical care and shaped their positive perceptions of intravenous iron therapy.

Because I learned about the dangers of anaemia, I was more scared to say no to the treatment when they told me that I was anaemic. I knew it would benefit my life. (IDI, Pregnant woman#14)

#### Ability to seek: peer support facilitated informed healthcare decision-making

Women also reported that peer support, particularly shared experiences from relatives or other pregnant women who had previously received intravenous iron, was influential in their decisions to seek care. Hearing positive outcomes reassured them, countered circulating myths and increased their confidence in pursuing treatment. These trusted testimonies motivated women to visit health facilities and follow through with recommended intravenous iron administration.

Oh yes! I did. I heard from a friend explaining that the intravenous iron was not appropriate. They said that the nurses take blood from pregnant women for other things. But I had a relative who had anaemia and was given IV iron. She told me how her condition had changed and encouraged me to receive the [intravenous iron] treatment, and I disregarded the rumours. (IDI, Pregnant woman#7)

In addition, increased health literacy fostered a sense of autonomy that further supported help-seeking. Women described feeling responsible for safeguarding their own health and that of their unborn children. This sense of agency empowered them to seek intravenous iron treatment independently, even when societal norms or family opinions discouraged them from doing so.

I considered myself lucky when I became aware of my anaemic condition. Myths and misconceptions could not stop me from prioritising my health. After all, my husband and friends could not find a solution to my problem. So, I did what was best for me. (IDI, Pregnant woman#7)

#### Ability to reach: social and financial support from family and husband

Pregnant women highlighted the importance of social and familial support in facilitating access to intravenous iron treatment. Many reported that having a caregiver, such as a mother, mother-in-law or husband, was instrumental in enabling them to reach health facilities for antenatal care. They noted that their husbands would accompany them to antenatal care visits on a motorbike or arrange transportation, such as hiring a ‘kabaza’ (bicycle taxi).

My husband was always available to accompany me for my antenatal care visits, and the day I was diagnosed with anaemia to receive intravenous iron, I was with him. (FGD1, Pregnant woman#2)

Pregnant women also reported that financial assistance, such as husbands’ provision of ‘yodyera kusikelo’ (pocket money), was a key motivator for them to attend antenatal care, where they would receive the support they needed for their pregnancy journey from healthcare providers, including intravenous iron.

Every time, I look forward to my scheduled visit. I benefit a lot. My husband gives me “yodyera kusikelo” (pocket money), making me a proud wife. (IDI, Pregnant woman#3)

#### Ability to engage: caregivers’ support; emotional and practical support from husband or mother/mother-in-law when receiving intravenous iron

Pregnant women emphasised the importance of emotional support from caregivers, such as husbands, mothers or mothers-in-law, in encouraging them to receive intravenous iron treatment. The reassurance and motivation from these key figures helped them overcome fears and doubts during the process and made them more confident in engaging with the intravenous iron intervention.

When my wife was found with low blood levels and was experiencing heavy dizziness and headache, I was worried because I knew she was at risk of losing her life and the child. When we were told about the IV iron intervention, I encouraged her to accept the treatment, and I was there during the administration. I encouraged her and assured her she would be fine. All we wanted was for her to feel better. (FGD2, Husband#3)

## Discussion

Using the PCAH framework, we identified several demand and supply barriers and facilitators affecting intravenous iron access and utilisation for the treatment of anaemia in pregnancy. Barriers included limited awareness of intravenous iron in the community, long travel distances from home to healthcare facilities, long waiting times to receive intravenous iron treatment, pregnant women’s non-compliance with appointments, myths and misconceptions about intravenous iron, cultural norms and inadequate social or financial support from husbands. Facilitators included access to clear information about anaemia and antenatal care services, peer support, the absence of pressure to receive intravenous iron, accessible antenatal care services with flexible appointments, perceived benefits of intravenous iron, healthcare providers’ interpersonal skills, better health literacy, social support from family or husbands and caregiver involvement. We demonstrated how supply and demand domains influence healthcare decisions and deepen our understanding of patients’ needs, preferences and experiences. This approach underscores the need for more effective, accessible and responsive health interventions for the target population.

### Limited awareness, structural and socio-cultural barriers: a multi-dimensional challenge to intravenous iron access and utilisation in pregnancy

#### Limited awareness

We identified a general lack of knowledge about anaemia and intravenous iron, driven by limited community awareness, as a key barrier to receiving intravenous iron. The limited awareness about intravenous iron created uncertainty, anxiety and hesitancy, discouraging some pregnant women from seeking intravenous iron treatment. In addition, the trial-related procedures, such as repeated blood sample collection from both mother and child and the collection of ‘nsengwa’, heightened fears among pregnant women and further reduced their willingness to accept intravenous iron. This finding aligns with a literature review of the primary barriers and facilitators to participation in clinical research, confirming that a lack of knowledge and understanding of new healthcare interventions influences negative attitudes towards accepting them.^31^ This suggests that knowledge in healthcare delivery is a fundamental aspect of successful implementation and a critical factor in building trust and promoting informed decision-making.^32^ The importance of targeted outreach education and effective open communication cannot be overstated, as they improve knowledge and address participants’ mistrust and fears.^31^ When healthcare providers provide clear, comprehensive information, pregnant women’s confidence in the healthcare system increases, enhancing their willingness to engage in interventions such as intravenous iron therapy. Improved awareness and understanding strengthen knowledge, promote timely care and support adherence to treatment.

#### Structural and socio-cultural barriers

Our findings demonstrated that the social, structural and socio-cultural barriers to intravenous iron access and utilisation are intertwined. For instance, the absence of continuous targeted community awareness campaigns about intravenous iron leads to increased cultural norms and social beliefs, such as myths and misconceptions about intravenous iron and behaviours of concealing a pregnancy. As a result, this creates fear and scepticism in pregnant women’s willingness to use intravenous iron. Likewise, long distances to the health facilities, lack of transportation, long waiting times and lack of social support from husbands not only discouraged women from accessing intravenous iron therapy and missing appointments but also contributed to delays in seeking treatment for anaemia in pregnancy. The findings on the barriers reported in this study mirror those of studies on the obstacles to the use of intravenous iron among pregnant women with anaemia in Nigeria,^33^ Malawi^34^ and Australia.^20^ Other studies have also reported similar barriers to access and use of antenatal care services,^35^,^39^ suggesting that challenges to intravenous iron use are embedded within broader structural and experiential barriers to antenatal care. These interconnected barriers for a pregnant woman living in rural areas of Malawi mean discouragement in timely reaching for healthcare services, especially for intravenous iron, which can only be administered in a health facility setting. Although access to antenatal care and the use of intravenous iron could face persistent barriers, pregnancy in low-resource settings represents a rare and critical moment when women engage with the health system.^40^ This unique window must not be wasted; it is an urgent call to dismantle these barriers and ensure women not only access but fully benefit from life-saving interventions (like intravenous iron) that can improve maternal and child health outcomes. Addressing socio-cultural perceptions and idioms will be crucial in a pregnant woman’s decision to seek care and adhere to treatment.

### Bridging gaps in access to and utilisation of intravenous iron: a balanced approach between demand and supply side enablers

Several interconnected domains facilitated access to and utilisation of intravenous iron. The availability of educational information about anaemia and intravenous iron services played a pivotal role in enabling pregnant women to recognise their healthcare needs and actively seek care. Their health literacy, the social value ascribed to their health and the degree of autonomy in making healthcare decisions influenced their decision to accept the intervention, despite the reported cultural and social factors. Malawi has made significant progress in antenatal care coverage, where about 97% of pregnant women access antenatal care.^41^ This highlights that pregnant women have adequate health literacy and understand the importance of attending antenatal care, prompting them to recognise the importance of intravenous iron therapy and make informed decisions about their health.

Pregnant women in our study consistently reported that promoting male involvement in antenatal care services would foster their acceptance of intravenous iron. The literature reveals that traditional notions of men as decision-makers and socio-cultural views on health remain vital, as men are considered the primary decision-makers in many households. Men still play a crucial role in providing financial support in their homes, highlighting their influence in shaping household dynamics and health decisions.^42^ A study in Somaliland found that pregnant women who were motivated and accompanied by their husbands to antenatal care were more likely to use these services than those who were not.^39^ By fostering shared responsibility between husband and wife for antenatal care, pregnancy can be reframed not solely as a woman’s reproductive roles and responsibilities but as a collaborative effort, thereby redefining the perception that men and women occupy different spaces over which they have authority. This will ultimately make it easy for pregnant women to decide and reach for healthcare interventions such as intravenous iron, therefore enhancing maternal and child health outcomes.

The goal of any health intervention is to align seamlessly with the patient’s needs, ensuring its relevance and effectiveness. Our study demonstrates that pregnant women perceived the intravenous iron intervention as an appropriate and efficacious treatment for anaemia. Moreover, evidence from individuals who had previously benefited from the intravenous iron intervention proved pivotal in fostering active engagement, as their firsthand experiences provided tangible proof of the treatment’s effectiveness. Women were impressed that, unlike oral iron tablets, intravenous iron works more rapidly, avoids gastrointestinal side effects and eliminates the burden of daily medication, making it a more patient-centred approach. These findings are consistent with a systematic review by Kumari *et al*, which highlighted that intravenous iron is associated with better compliance, fewer gastrointestinal side effects and greater patient satisfaction compared with oral iron.^43^ This perceived fit between the intervention and their healthcare needs significantly enhances the acceptability of the intervention, demonstrating the profound impact of compelling evidence in validating the appropriateness of the intervention and ensuring active engagement.

Collectively, these findings indicate that addressing factors that affect the uptake of health interventions, such as intravenous iron, improves the acceptance and use of these interventions for managing anaemia in pregnant women.

### Strengths and limitations

To our knowledge, this is the first study to apply the PCAH framework to assess factors influencing access to and use of intravenous iron for anaemia treatment among pregnant women in Malawi. This approach provides a comprehensive understanding of the patient’s journey. By examining the intervention from the end user’s perspective, we gained critical insights into how to enhance intravenous iron uptake. Including both participants and withdrawals from the REVAMP-TT trial, along with their caregivers, strengthened our findings and reduced bias. The use of IDIs and FGDs allowed for an in-depth exploration of social support during pregnancy. However, we did not capture perspectives from women who declined to participate in the trial or received oral iron. Also, we did not explore the influence of traditional or religious healers on women’s decisions about intravenous iron. Additionally, while this study does not address the affordability and ability-to-seek domains of the PCAH framework, a separate paper will examine the cost implications. The influence of financial reimbursements on positive responses to intravenous iron should also be considered in future evaluations.

## Conclusion

Anaemia in pregnancy is a common condition disproportionately affecting women who live in LMICs. Our study examined the demand and supply barriers and facilitators influencing access to and use of intravenous iron for the treatment of anaemia in pregnancy across health facilities in Malawi. The results highlight both the potential of using intravenous iron for treating anaemia in pregnancy and the contextual factors shaping its uptake. Key barriers included limited awareness, long travel distance to the clinic, prevailing cultural norms and myths and misconceptions, and lack of social support. Addressing these barriers requires improving flexible and accessible service delivery, enhancing health literacy and awareness, fostering supportive provider-patient interactions and strengthening social support networks to encourage pregnant women to seek essential antenatal care services. Strengthening the health system’s capacity through training for healthcare providers, improving infrastructure and road networks and enhancing community education is crucial for optimising the utilisation of health interventions and ensuring they are accessible and responsive to the diverse needs of end users. These efforts will ultimately improve maternal and child health outcomes and promote equity in healthcare access.

## Supplementary material

10.1136/bmjgh-2025-019886 online supplemental file 1

## Data Availability

Data are available upon reasonable request.
