# Supplementary material for: Factors affecting access to and utilisation of intravenous iron to treat anaemia in pregnancy in Zomba, Malawi: a qualitative study
Source: BMJ Glob Health. 2026 May 7;11(5):e019886. doi: 10.1136/bmjgh-2025-019886 (PMC13157774; doi:10.1136/bmjgh-2025-019886)
Supplement: online supplemental file 1 [file bmjgh-11-5-s001.docx]

### BMJ Global Health Author Reflexivity Statement

Adapted from Morton, B., Vercueil, A., Masekela, R., Heinz, E., Reimer, L., Saleh, S., Kalinga, C., Seekles, M., Biccard, B., Chakaya, J., Abimbola, S., Obasi, A. and Oriyo, N. (2022), Consensus statement on measures to promote equitable authorship in the publication of research from international partnerships. Anaesthesia, 77: 264-276. <https://doi.org/10.1111/anae.15597>

| **Study conceptualisation** | |
| --- | --- |
| 1. How does this study address local research and policy priorities? | This study responds directly to Malawi’s maternal health priorities by addressing the high burden of anaemia in pregnancy and the need for effective, acceptable, and scalable treatment options. Intravenous iron has been commonly used in the high-income countries, yet evidence on access, acceptability, in low-middle-income countries is limited. The study generated context-specific evidence to inform policy decisions on integrating IV iron within the public health systemin Malawi |
| 1. How were local researchers involved in study design? | Local researchers based at Kamuzu University of Health Sciences led the conceptualisation of the study, development of the research questions, study design, and data collection tools. The study design was informed by co-designing implementation strategies with healthcare providers and community members for realities and contexts to ensure relevance. |
| **Research management** | |
| 1. How has funding been used to support the local research team(s)? | Funding supported tuition fees for the PhD student, training in qualitative research methods, data collection activities, transcription, and stakeholder and community engagement meetings. |
| **Data acquisition and analysis** | |
| 1. How are research staff who conducted data collection acknowledged? | Research assistants are formally acknowledged in the manuscript and were integral to data collection and transcription. Their contextual knowledge contributed to the quality and depth of the data collected. |
| 1. How have members of the research partnership been provided with access to study data? | All members of the research partnership were granted access to anonymised transcripts and analytical memos through secure data-sharing platforms. Regular meetings were held to discuss emerging findings and interpretations. |
| 1. How were data used to develop analytical skills within the partnership? | Joint data analysis workshops were conducted, during which local researchers were trained in developing codebook, qualitative coding, framework analysis, and interpretation. The PhD student actively led in coding and theme development under mentorship from senior qualitative researchers. |
| **Data interpretation** | |
| 1. How have research partners collaborated in interpreting study data? | Interpretation of findings was conducted collaboratively through iterative team discussions. Local researchers played a central role in contextualising findings within the Malawian health system. |
| **Drafting and revising for intellectual content** | |
| 1. How were research partners supported to develop writing skills? | The PhD student was supported through structured writing mentorship, iterative feedback on manuscript drafts, and leading in the submission of the manuscript and responding to peer-review comments. Lead authorship roles was assigned to the LMIC PhD student. |
| 1. How will research products be shared to address local needs? | Findings will be disseminated through policy brief, stakeholder meetings with the Ministry of Health, district health teams, and professional bodies, as well as community feedback sessions. These outputs are designed to support evidence-informed decision-making and implementation planning. |
| **Authorship** | |
| 1. How is the leadership, contribution and ownership of this work by LMIC researchers recognised within the authorship? | LMIC researchers occupy lead and senior authorship positions, including first and guarantor roles. The lead author is a Malawi-based PhD student, reflecting local leadership, substantial intellectual contribution, and ownership of the research agenda |
| 1. How have early career researchers across the partnership been included within the authorship team? | Early-career researchers were included as co-authors based on their contributions to review of data collection tools, analysis, and manuscript development. |
| 1. How has gender balance been addressed within the authorship? | The authorship team is entirely female, comprising women researchers across junior and senior levels, with women occupying leadership and lead authorship positions. |
| **Training** | |
| 1. How has the project contributed to training of LMIC researchers? | The project provided hands-on training and experience in qualitative research methods, reflexivity, data analysis, scientific writing, and research governance, contributing to long-term capacity strengthening among LMIC researchers. |
| **Infrastructure** | |
| 1. How has the project contributed to improvements in local infrastructure? | The project strengthened local research and implementation infrastructure through improved collaboration between academic institutions, health facilities, community structures, and policymakers. Institutional capacity was enhanced via data collection tools, transcription resources, and secure data management systems, while engagement with community gatekeepers and policymakers strengthened platforms for sustained community participation and evidence-informed decision-making. |
| **Governance** | |
| 1. What safeguarding procedures were used to protect local study participants and researchers? | Ethical approval was obtained from the College of Medicine Research Ethics Committee and Zomba district health management Committee. Written informed consent was obtained from all participants. Safeguarding procedures included confidentiality protections, secure data storage, de-identification of transcripts interviewer training on ethical conduct, and referral mechanisms for participants requiring further management. |
